# Endothelial dysfunction in obese non-hypertensive children without evidence of sleep disordered breathing

**DOI:** 10.1186/1471-2431-10-8

**Published:** 2010-02-15

**Authors:** Rakesh Bhattacharjee, Wadha H Alotaibi, Leila Kheirandish-Gozal, Oscar Sans Capdevila, David Gozal

**Affiliations:** 1 Division of Pediatric Sleep Medicine, Department of Pediatrics, University of Louisville, Kentucky, USA; 2 Kosair Children's Hospital Research Institute, Louisville, Kentucky, USA

## Abstract

**Background:**

Endothelial dysfunction is a complication of both obesity and obstructive sleep apnea syndrome (OSAS), the latter being highly prevalent among obese children. It is unknown whether obesity causes endothelial dysfunction in children in the absence of OSAS. This study examines endothelial function in obese and non-obese children without OSAS.

**Methods:**

Pre-pubertal non-hypertensive children were recruited. Endothelial function was assessed in a morning fasted state, using a modified hyperemic test involving cuff-induced occlusion of the radial and ulnar arteries. The absence of OSAS was confirmed by overnight polysomnography. Anthropometry was also performed.

**Results:**

55 obese children (mean age 8.6 ± 1.4 years, mean BMI z-score: 2.3 ± 0.3) were compared to 50 non-obese children (mean age 8.0 ± 1.6 years, mean BMI z-score 0.3 ± 0.9). Significant delays to peak capillary reperfusion after occlusion release occurred in obese compared to non-obese children (45.3 ± 21.9 sec *vs*. 31.5 ± 14.1 sec, p < 0.01), but no differences in the magnitude of hyperemia emerged. Time to peak reperfusion and percentage of body fat were positively correlated (r = 0.365, p < 0.01).

**Conclusions:**

Our findings confirm that endothelial dysfunction occurs early in life in obese children, even in the absence of OSAS. Thus, mechanisms underlying endothelial dysfunction in pediatric obesity are operational in the absence of sleep-disordered breathing.

## Background

The obesity epidemic has raised awareness to the potential and far-reaching adverse consequences associated with being overweight. Indeed, obesity has been associated with an increase in the prevalence of diabetes mellitus, dyslipidemia, hypertension, and potentially leads to the development of the metabolic syndrome and cardiovascular disease [1,2]. In addition, obesity markedly increases the risk for obstructive sleep apnea syndrome (OSAS) [3-6].

Obesity in children has nearly doubled over the past 2 decades in the United States, with approximately 15% of all children fulfilling obesity criteria, i.e., a body mass index (BMI) over the 95^th ^percentile on standard growth curves assembled by the Centers for Disease and Control (CDC) [7]. As a consequence of the increasing rates of childhood obesity, and the above-listed obesity-associated co-morbidities, the global life expectancy in the United States is expected to decline, [8-10] and has led to the American Heart Association as well as the American Diabetes Associations both to re-classify obesity as a 'major, modifiable risk factor' for cardiovascular disease [11].

There is ample evidence suggesting that the complications of obesity in adults begin in early childhood. Hypercholesterolemia, dyslipidemia, [9,12,13] insulin resistance, and type 2 diabetes mellitus [14-16] have been all described as significant co-morbidities associated with pediatric obesity. Likely, the constellation of these metabolic derangements accrues a significant risk for the development of cardiovascular disease during childhood. Furthermore, although systemic hypertension in children is rare with an estimated prevalence at 2-5%, [17] obesity has been shown to increase the risk of hypertension in children [18,19].

In addition to cardiovascular morbidity, obesity has been linked to the development of OSAS in children, a condition in which intermittent occlusion of the upper airway during sleep leads to recurrent oxyhemoglobin desaturations, elevated carbon dioxide levels, sleep fragmentation, and reduced sleep efficiency [4-6,20]. Among other morbidities, OSAS independently elevates the risk for systemic hypertension and cardiovascular disease [21]. Recent work from our laboratory has shown that endothelial dysfunction, an early marker of cardiovascular disease, which was assessed by a modified hyperemic test after cuff-induced occlusion of the brachial artery, was also present among non-obese 6-9-year-old children who were polysomnographically diagnosed with OSAS, and that the abnormal post-occlusive reperfusion responses were reversed in most children by adequate and effective treatment of their underlying OSAS [22].

Obesity in children has also been shown to induce endothelial dysfunction [23,24]. However, although the latter studies showed significant associations between body fat and dyslipidemia, insulin resistance, and inflammation, all of which could contribute to endothelial dysfunction, none of these studies determined whether OSAS was concomitantly present. Thus, there is no certainty as to whether the abnormalities in endothelial function described in obese children were specifically linked to the obese state or to the concurrent yet unknown presence of OSAS.

The purpose of the current study was to investigate the impact of obesity on endothelial dysfunction in children by careful exclusion of children with OSAS.

## Methods

The study was approved by the University of Louisville and Kosair Children's Hospital institutional review boards. Informed consent from the parent or legal custodian was obtained, and minor assent (for children ages 7 and older) was administered and obtained as well. Consecutive healthy pre-pubertal children (ages 4-12 years) participating in a sleep and neurocognitive research study at the University of Louisville Pediatric Sleep Medicine Center were recruited to investigate endothelial function. Subjects were recruited from November 2007 until September 2008. All participants underwent baseline overnight polysomnography and a fasting blood draw in the morning.

### Anthropometry

Children were weighed using the InBody 320 scale (Biospace, CA), which employs the Direct Segmental Multi-frequency Bioelectrical Impedance Analysis method, and therefore enables assessment of percent body fat and determination of individual impedance indices for all limbs. This feature allows for computation of an estimate of visceral fat. Height (to 0.1 cm) was measured with a stadiometer (Holtain, Crymych, UK). BMI was calculated and BMI z-score was computed using CDC 2000 growth standards http://www.cdc.gov/growthcharts and online software http://www.cdc.gov/epiinfo. A BMI z-score >1.65 (>95^th ^percentile) was considered as fulfilling obese criteria.

### Sphygmomanometry

All children had arterial blood pressure measured non-invasively using an automated mercury sphygmomanometer (Welch Allyn, NY) at the brachial artery using a guidelines-defined appropriate cuff size on the non-dominant arm [25]. Two consecutive blood pressure measurements were made while children lay supine with the head of the bed elevated to 45°. A non-standard posture of blood pressure measurements was chosen in order to mimic the posture utilized during endothelial function testing, which was both comfortable for children and best minimized arm motion artifact during measurement. Blood pressure measurements were made in the evening prior to commencement of nocturnal polysomnography. Systolic and diastolic blood pressure indices were calculated by dividing the average systolic and diastolic pressure by the respective 95^th ^percentile for blood pressure http://www.nhlbi.nih.gov/guidelines/hypertension/child_tbl.htm computed for age, gender and height. Hypertension was defined by a systolic or diastolic blood pressure index exceeding 1.

### Overnight Polysomnography (PSG)

PSG was conducted and scored as previously reported [26].

### Endothelial Function

Endothelial function was assessed using a modified hyperemic test after cuff-induced occlusion of the radial and ulnar arteries by placing the cuff over the wrist. All testing was performed at awakening to ensure that children were in a fasted state. A laser Doppler sensor (Periflux 5000 System integrated with the PF 5050 Pressure Unit, Perimed, Järfälla, Sweden) was applied over the volar aspect of the hand at the 1^st ^finger distal metacarpal surface and the hand was gently immobilized. This site was chosen as an area in order to minimize the effects of motion artifact and was also found to have a density of skin capillary blood flow that was of appropriate magnitude for detection. Again, children lay supine with the head of the bed elevated 45°. Once cutaneous blood flow over the area became stable, the pressure within an inflatable cuff placed at the forearm and connected to a computer-controlled manometer was raised to 200 mmHg for 60 sec during which blood flow was reduced to undetectable levels. An occlusion time of 60 seconds was chosen in order to minimize discomfort for the child. Using a computer controlled pressure release to allow for consistent deflation times, the cuff was rapidly deflated and the laser Doppler measured hyperemic responses. The maneuver was repeated twice within 10 min with at least 2 minutes separating both trials to ensure a return to baseline perfusion. The average of both maneuvers was then computed for subsequent analyses. Laser Doppler determines the magnitude of perfusion at rest, at occlusion and post occlusion. While detection of microvascular perfusion varies by child according to density of capillary blood vessels, thickness of skin, etc., all measurements are extrapolated to baseline perfusion before cuff occlusion, and analysis of reperfusion kinetics is based on time measurements. Commercially available software (Perimed, Järfälla, Sweden) allowed for unbiased estimates of the time to peak regional blood flow response post occlusion release, which is considered representative of the post-occlusion hyperemic response, an index of endothelial function [27]. The mean intra-individual coefficient of variability of the time to peak flow was 12.8% for all trials. When the time to peak flow in any of the subjects, in the 2 trials differed by >25%, the test was repeated a 3^rd ^time, and the 2 fastest times to peak reperfusion were retained and averaged. Only 4 of the subjects in the current study required a 3^rd ^trial

### Blood Tests

Fasting blood samples were drawn by venipuncture in the morning immediately after endothelial function testing and nocturnal polysomnography. Blood was sent for routine laboratory testing including high sensitivity C-reactive protein, glucose, insulin, and serum lipid concentrations, using standard laboratory techniques.

### Exclusion Criteria

All hypertensive children (with either a systolic or diastolic blood pressure index > 1) or using anti-hypertensive therapies were excluded. Furthermore, children with diabetes (fasting serum glucose ≥ 120 mg/dL), with a craniofacial, neuromuscular or defined genetic syndrome, and children on chronic anti-inflammatory therapy, or with any known acute or chronic illness were excluded. Children using sympathomometic agents such as psychostimulants were excluded. Finally, patients found to snore or have OSAS during the PSG, i.e, obstructive apnea hypopnea index (OAHI) ≥ 1.5 events/hour TST, were excluded from the analysis.

### Data Analysis

Results are presented as means ± SD, unless stated otherwise. All numerical data were subjected to statistical analysis using independent Student t tests or analysis of variance followed by post-hoc tests as appropriate. No variance stabilizing transformations were undertaken. A 2-tailed p < 0.05 was considered to define statistical significance. Chi square analysis was performed on categorical data concerning demographic characteristics of obese and non-obese groups.

All methods outlined in this study were approved by the University of Louisville Human Research Committee.

## Results

In total, 161 children fitting initial inclusion criteria were recruited. Of these, 49 children were found to have OSAS with OAHI > 1.5/hrTST and were therefore excluded. Six additional children were found to be mildly hypertensive (systolic blood pressure index > 1), and were also excluded from the study. Of the remaining 105 eligible children, 55 children were obese and the remaining 50 were designated into the non-obese group. The mean age of non-obese and obese groups was similar (8.0 ± 1.6 years compared to 8.6 ± 1.4 years, respectively; p - not significant). Gender and ethnic distribution were also similar across both groups (Table 1). Seven children in the obese group and 7 children in non-obese group had parents with systemic essential hypertension or with early onset cardiovascular disease (before age 50).

**Table 1:** Demographic Characteristics of Non-Obese and Obese Cohort of Pre-Pubertal Children

		Non-Obese (BMI z < 1.65)	Obese (BMI z > 1.65)	p value
**Number of Subjects**		50	55	

**Age**	**years**	8.0 ± 1.6	8.6 ± 1.4	NS

**Gender**	**Males**	30	27	NS

	**Females**	20	28	NS

**Race**	**White Non Hispanic**	33	30	NS

	**African American**	12	22	NS

	**Hispanic**	1	0	NS

	**Biracial**	2	3	NS

	**Other**	2	0	NS

**Family History of Cardiovascular Disease**		7	7	NS

**BMI**	**(kg/m2)**	16.8 ± 1.9	26.7 ± 4.7	p < 0.01

**BMI z score**		0.3 ± 0.9	2.3 ± 0.3	p < 0.01

**Lean Body Mass**	**(kg)**	22.8 ± 4.0	31.2 ± 5.9	p < 0.01

**Body Fat Mass**	**(kg)**	4.8 ± 2.9	21.5 ± 8.1	p < 0.01

**Dry Lean Mass**	**(kg)**	6.4 ± 1.1	8.7 ± 1.6	p < 0.01

**Percent Body Fat**	**(%)**	16.2 ± 7.0	39.2 ± 4.5	p < 0.01

**Mean Systolic Blood Pressure**	**(mmHg)**	97.7 ± 7.5	111.6 ± 8.4	p < 0.01

**Systolic Blood Pressure Index**		0.85 ± 0.06	0.94 ± 0.07	p < 0.01

**Mean Diastolic Blood Pressure**	**(mmHg)**	59.7 ± 6.4	62.3 ± 7.8	NS

**Diastolic Blood Pressure Index**		0.78 ± 0.08	0.80 ± 0.10	NS

Anthropometric assessments revealed significant differences in lean body mass, body fat mass, dry lean mass and percent body fat across the 2 groups (Table 1). Percent body fat was 39.2% in obese children compared to 16.2% in non-obese children (p < 0.01; Table 1). Obese children also exhibited significantly higher impedances in all limbs and in the trunk, indicative of increased visceral fat (p < 0.01; data not shown).

Overnight polysomnographic findings were similar between both groups and within the normal values previously reported for a healthy pediatric population(OAHI (events/hr): 0.8 ± 0.5 in the nonobese group vs. 0.9 ± 0.5, in the obese group) [28]. Apart from a significant, albeit minor increase in the percentage of wake time after sleep onset in the obese group, all polysomnographic variables were not significantly different between the obese and non-obese cohorts (Additional file 1, Table S4).

Blood pressure measurements, while not fulfilling any of the criteria for hypertension in the 105 children included in the present study, revealed that obese children had significantly higher mean systolic blood pressures (111.6 ± 8.4 vs. 97.7 ± 7.5 mmHg, p < 0.01) and systolic blood pressure indices (0.94 ± 0.06 vs. 0.85 ± 0.06, p < 0.01) compared to non-obese children. There were no differences in either diastolic blood pressure or diastolic blood pressure indices between both groups.

Metabolic profiles revealed no significant differences in either fasting serum cholesterol, apolipoprotein B, or triglyceride levels (Table 2), although triglyceride levels trended towards higher levels in obese children compared to non-obese children (p = 0.1) Fasting glucose or insulin levels were similar in the 2 groups. While serum levels of high sensitivity C-reactive protein (CRP) were not significantly different, there appeared to be a slight elevation in CRP in the obese group.

**Table 2:** Blood Metabolic Profiles of Non-Obese and Obese Cohort of Pre-Pubertal Children

		Non-Obese (BMI z < 1.65)	Obese (BMI z > 1.65)	p value
**Triglycerides**	(mg/dL)	73.2 ± 44.5	87.0 ± 33.4	NS

**Cholesterol**	(mg/dL)	163.5 ± 24.9	164.0 ± 28.9	NS

**HDL**	(mg/dL)	53.8 ± 11.4	50.0 ± 10.0	NS

**LDL**	(mg/dL)	95.1 ± 21.2	96.6 ± 24.1	NS

**Glucose**	(mg/dL)	73.5 ± 22.8	79.0 ± 11.4	NS

**Insulin**	(mIU/mL)	7.9 ± 11.6	11.0 ± 6.2	NS

**CRP**	(mg/L)	1.1 ± 1.7	2.8 ± 5.1	NS

**Apo-B**	(mg/L)	74.2 ± 16.5	77.2 ± 18.9	NS

Legend -- HDL -- high density lipoprotein, LDL -- low density lipoportein, CRP -- C-reactive protein, Apo-B -- apolipoprotein B

### Endothelial Function Testing

**Figure 1:**
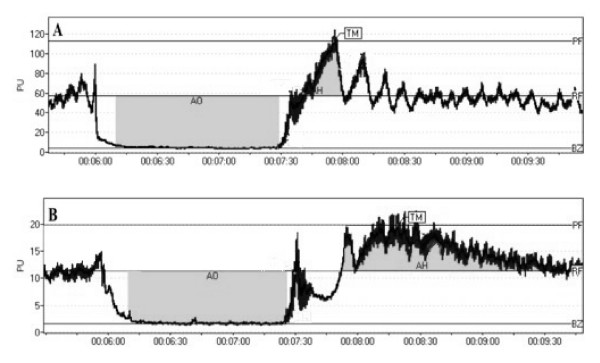
**Cuff Occlusion Testing in a Non-obese (A) and an Obese (B) child**. PU -- perfusion units, PF -- peak flow, RF -- rest flow, BZ -- biological zero, AO -- area of occlusion, AH -- area of hyperemia, TM -- time to peak flow following occlusion.

Laser-Doppler analysis of cutaneous blood flow did not reveal any significant differences in resting blood flow between both groups. Release of occlusion did not reveal any significant differences in the magnitude of peak flow after release of occlusion. However, the time to attain peak reperfusion flow in obese children was significantly delayed in comparison to non-obese children (45.3 ± 21.9 vs. 31.5 ± 14.1 s, p < 0.01; Table 3, Figure 1).

**Table 3:** Perfusion Kinetic Measures of Endothelial Function in Non-Obese and Obese Cohort of Pre-Pubertal Children

		Non-Obese (BMI z < 1.65)	Obese (BMI z > 1.65)	p value
**Rest Flow**	(PU)	21.2 ± 11.9	22.5 ± 12.6	NS

**Biological Zero (BZ)**	(PU)	3.8 ± 2.1	3.4 ± 1.7	NS

**Peak Flow (PF)**	(PU)	43.7 ± 25.1	44.2 ± 20.3	NS

**Time to Peak Flow**	(s)	31.5 ± 14.1	45.3 ± 21.9	p < 0.01

Legend -- PU -- perfusion units

**Figure 2:**
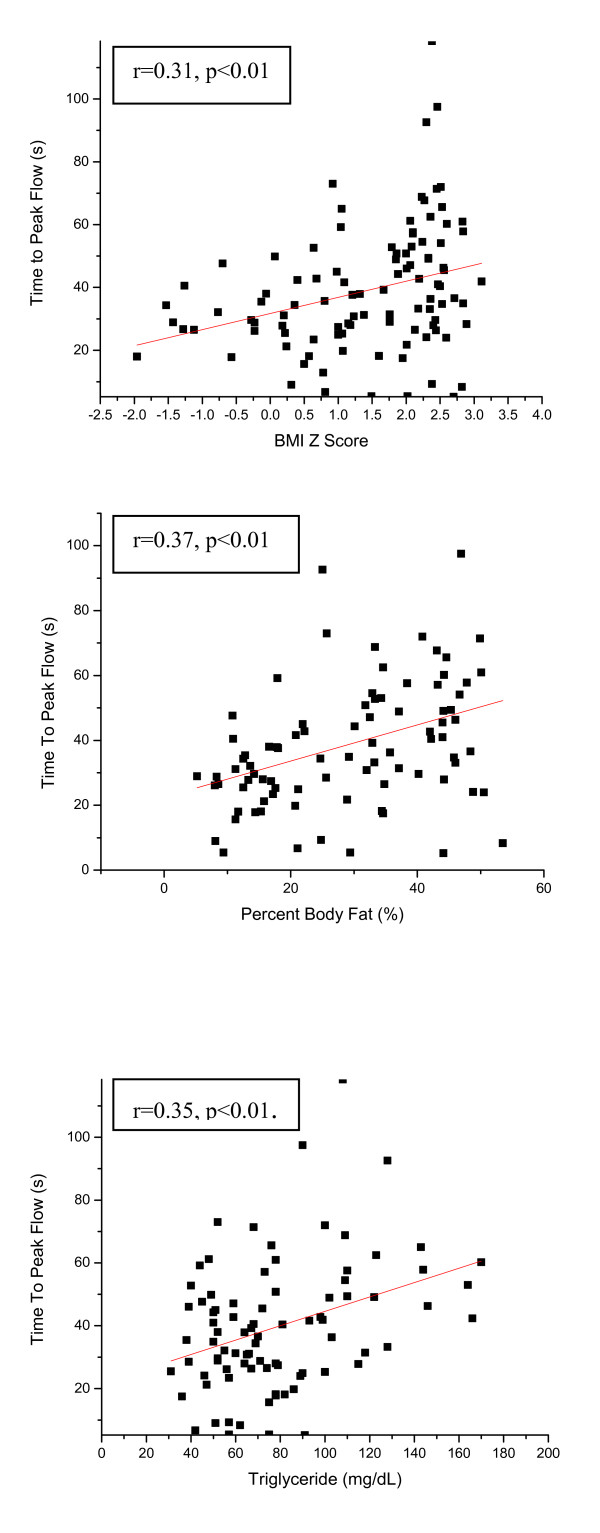
**(Top) Relationship between BMI Z-score and Time to Reperfusion Peak Flow (r = 0.31, p < 0.01) (Middle) Relationship between Body Fat Percentage and Time to Reperfusion Peak Flow (r = 0.37, p < 0.01) (Bottom) Relationship between Serum Triglyceride Concentration and Time to Reperfusion Peak Flow (r = 0.35, p < 0.01)**.

The potential impact of obesity on endothelial function is further illustrated in Figure 2. Significant positive associations emerged between BMI z-score (r = 0.31, p < 0.01), and between percent body fat (r = 0.37, p < 0.01) and time to peak flow, suggesting that increasing severity of obesity in children leads to increased delays in reperfusion responses. Similarly, a positive correlation between serum triglyceride levels and time to peak flow was found (figure 2), thereby linking hyperlipidemia to endothelial dysfunction (r = 0.35, p < 0.01). Using stepwise logistic regression, a model was constructed, whereby BMI z-score accounted for 24.7% of the variance in time to peak flow, with additional significant, albeit minor contributions by visceral fat (5.2%), but not by lipid serum concentrations.

## Discussion

To the best of our knowledge, the present study is the first to demonstrate the presence of endothelial dysfunction in obese, non-hypertensive pre-pubertal children in the absence of concomitant obstructive sleep apnea syndrome. Delays in vascular reperfusion kinetics suggest that the integrity of the vasculature that is critical for adequate responses to vasodilatory stimuli, such as those operating in post-ischemic recovery responses, is significantly compromised in obese children, and that such impairments are correlated to the degree of dyslipidemia and percent body fat.

Before discussing the potential implications of our findings, several methodological issues deserve comment. First, by using a rather holistic diagnostic approach, this study successfully recruited a large cohort of obese children with no evidence of OSAS or any other form of sleep disordered breathing. Hence, one can then more confidently ascribe the aberrations in vascular function identified in these obese children to obesity itself, and clearly rule out that these abnormalities may have been imposed by some of the systemic metabolic and oxidative stress alterations associated with OSAS. Further, 7 children in each of the 2 groups had a significant family history of early onset hypertension or premature cardiovascular disease, and therefore, any confounding effects of any known familial factors contributing to early onset cardiovascular disease in the obese group are unlikely to have played a role in the differences in reperfusion kinetics between obese and non-obese children. Thirdly, only non-hypertensive children were included in the study, further restricting the potential confounder effects of hypertension on the impact of obesity on vascular function.

As previously shown by other studies, albeit without ascertaining on the concurrent presence of OSAS, [23,24,29,30] and now further confirmed by the current study, the presence of endothelial dysfunction in obese children implies that early changes leading to atherosclerosis may be already actively operational. The endothelial surface of the vascular wall is the frontline of vascular functional integrity, and is also instrumental in the early formation of the atherogenic plaques [31]. While paracrine functioning of healthy vascular endothelium contributes to protection against such plaque formation, our identification of abnormal endothelial function would suggest that these homeostatic processes are impaired, thereby posing the risk of early onset atherosclerosis in pre-pubertal children. However, we should point out that delineation of endothelial dysfunction by the current cutaneous vascular reperfusion test used herein differs from flow mediated reperfusion tests using Doppler technology assessment of a muscular artery (e.g., brachial artery), and that therefore our current findings can not be extrapolated to other measurement techniques. Further, the decision to minimize the duration of the occlusion such as to prevent discomfort and motion in very young children may limit generalizability of the technique.

We have previously shown that OSAS is associated with reversible injury to the vascular endothelium in non-obese children and that these pathological processes were reversible in the vast majority of children upon effective treatment of OSAS [22]. Furthermore, the presence of a strong family history of early cardiovascular disease in a subset of ~20% of these children was associated with lack of reversibility of endothelial hyperemic delays at 6 months after treatment. In the present study, we excluded any child with altered sleep-associated breathing abnormalities patterns, and, thus, our current findings more definitively confirm that endothelial dysfunction associated with obesity occurs independently of OSAS. Nevertheless, it is likely that both obesity- and OSAS-associated mechanisms of vascular dysfunction overlap, and may in fact synergistically interact and converge; such as to induce amplified injury to the vascular endothelium through possible shared inflammatory, vasoconstrictor and pro-atherothrombotic pathways [32].

While obese children studied herein were not hypertensive, and yet, obesity was associated with increases in systolic blood pressure indices, in the absence of similar changes in diastolic measures. Although current guidelines would suggest that treatment with anti-hypertensive agents is not indicated in these children, [25] the cumulative evidence of elevated systolic blood pressure indices and endothelial dysfunction in the context of obesity would provide further inferences as to the presence of underlying premature cardiovascular disease in obese, non-hypertensive, non-apneic, pre-pubertal children. It is possible that future studies in such children may lead to a change in treatment guidelines, which currently advocate treatment of only those children with blood pressure exceeding the 95^th ^percentile, especially considering the concurrent presence of endothelial dysfunction.

Of note, while this study did not address the reversibility of endothelial dysfunction in this obese cohort, several reports have shown that adequate dietary modification combined with exercise can return endothelial function to control levels in obese children, [33,34] and may therefore suffice.

## Conclusions

Cardiovascular morbidity is a well-ascribed complication of obesity and OSAS in children. In this report, we show that abnormal endothelial function is significantly associated with obesity in otherwise healthy, non-hypertensive, pre-pubertal children who do not have any polysomnographic evidence of OSAS. Further studies are warranted to assess the beneficial effect of obesity reversal interventions in this population, and to determine the potential interactions between OSAS and obesity in the context of the cardiovascular morbidity associated with these 2 highly prevalent conditions.

## List of Abbreviations

BMI: body-mass-index; CRP: high sensitivity C reactive protein; OAHI: obstructive apnea hypopnea index; OSAS: obstructive sleep apnea syndrome; PSG: overnight polysomnography; SpO_2_: Arterial pulse oxygen saturation; TST: total sleep time.

## Conflict of interests

The authors have no conflict of interest to declare in relation to this manuscript.

***Financial Disclosures***: DG is on the National Speaker Bureau for Merck Co and has given lectures for honoraria on asthma and allergy.

## Authors' contributions

RB has made substantial contributions to conception and design, or acquisition of data by conducting polysomnography scoring, endothelial function testing and data acquisition, and analysis and interpretation of data; he was also involved in drafting the manuscript or revising it critically for important intellectual content; and has given final approval of the version to be published. WHA has made substantial contributions to acquisition of data by conducting polysomnography scoring, endothelial function testing; and has given final approval of the version to be published. LKG has made substantial contributions to conception and design, analysis and interpretation of data; and has given final approval of the version to be published. OSC has made substantial contributions acquisition of data by conducting polysomnography scoring, and has given final approval of the version to be published. DG has conceived the study design, supervision of the study personnel, analysis and interpretation of data; has been involved in drafting the manuscript or revising it critically for important intellectual content, and has given final approval of the version to be published.

## Pre-publication history

The pre-publication history for this paper can be accessed here:

http://www.biomedcentral.com/1471-2431/10/8/prepub

## Supplementary Material

Additional file 1 **Table S4**. Polysomnographic Characteristics of Non-Obese and Obese Cohort of Pre-Pubertal Children. Click here for file
