# Supplementary material for: Endothelial dysfunction in obese non-hypertensive children without evidence of sleep disordered breathing
Source: BMC Pediatr. 2010 Feb 15;10:8. doi: 10.1186/1471-2431-10-8 (PMC2829007; doi:10.1186/1471-2431-10-8)
Supplement: Additional file 1 — Table S4. Polysomnographic Characteristics of Non-Obese and Obese Cohort of Pre-Pubertal Children. [file 1471-2431-10-8-S1.DOC]

**Table S4– Polysomnographic Characteristics of Non-Obese and Obese Cohort of Pre-Pubertal Children**

|  | **Units** | **Non-Obese (BMI z< 1.65)** | **Obese (BMI z > 1.65)** | **p value** |
| --- | --- | --- | --- | --- |
| **Total Sleep Time** | (min) | 458.7±49.4 | 448.3±48.3 | NS |
| **Sleep Efficiency** | (%) | 88.9±7.2 | 86.1±8.3 | NS |
| **Sleep Onset Latency** | (min) | 23.7±23.2 | 31.2±25.7 | NS |
| **REM Onset Latency** | (min) | 163.7±69.2 | 183.7±70.3 | NS |
| **Awakenings** | (n) | 10.3±5.5 | 11.6±5.8 | NS |
| **WASO** | (%TST) | 3.7±8.1 | 4.9±11.9 | p<0.05 |
| **Stage 1** | (%TST) | 3.6±3.2 | 4.5±3.4 | NS |
| **Stage 2** | (%TST) | 42.9±9.6 | 42.3±7.4 | NS |
| **Stage 3** | (%TST) | 10.2±7.4 | 10.1±7.3 | NS |
| **Stage 4** | (%TST) | 22.4±8.3 | 21.1±9.0 | NS |
| **Stage REM** | (%TST) | 17.2±6.1 | 17.1±5.2 | NS |
| **SAI** | (/hr TST) | 12.3±7.1 | 12.4±9.7 | NS |
| **RAI** | (/hr TST) | 0.5±0.5 | 0.5±0.7 | NS |
| **PLMI** | (/hr TST) | 8.2±7.5 | 7.0±2.3 | NS |
| **PLMAI** | (/hr TST) | 0.4±0.7 | 0.3±0.5 | NS |
| **OAHI** | (/hr TST) | 0.8±0.5 | 0.9±0.5 | NS |
| **O_2_ Saturation Nadir** | (%) | 91.9±2.8 | 90.3±5.3 | NS |
| **Mean ETCO_2_** | (mmHg) | 42.2±3.1 | 42.7±3.9 | NS |
| **Peak ETCO_2_** | (mmHg) | 50.7±3.8 | 50.1±4.4 | NS |

Legend: WASO – wake after sleep onset, REM – rapid eye movement, TST – total sleep time, SAI – spontaneous arousal index, RAI – respiratory arousal index, PLMI – periodic limb movement index, PLMAI – periodic limb movement with arousal index, OAHI – obstructive apnea hypopnea index, ETCO_2_ – end tidal carbon dioxide
